# “In a situation of rescuing life”: meanings given to diabetes symptoms and care-seeking practices among adults in Southeastern Tanzania: a qualitative inquiry

**DOI:** 10.1186/s12889-015-1504-0

**Published:** 2015-03-07

**Authors:** Emmy Metta, Ajay Bailey, Flora Kessy, Eveline Geubbels, Inge Hutter, Hinke Haisma

**Affiliations:** Ifakara Health Institute, Box 78373, Dar es Salaam, Tanzania; Population Research Centre, Faculty of Spatial Sciences, University of Groningen, Landleven 1, 9742 AK Groningen, The Netherlands; Mzumbe University, Box 20226, Dar es Salaam, Tanzania

**Keywords:** Diabetes, Cultural meanings, Traditional healers, Witchcraft, Rural

## Abstract

**Background:**

Diabetes mellitus is an emerging public health problem in Tanzania. For the community and the health system to respond adequately to this problem, it is important that we understand the meanings given to its symptoms, and the care-seeking practices of individuals.

**Methods:**

To explore collective views on the meanings given to diabetes symptoms, we conducted nine focus group discussions with adult diabetes patients and members of the general community. To gain a better understanding of how the meanings in the community inform the care-seeking practices of individuals, 19 in-depth interviews were conducted with diabetes patients. The data were analyzed using principles of grounded theory and applying cultural schema theory as a deductive framework.

**Results:**

In the communities and among the patients, knowledge and awareness of diabetes are limited. Both people with diabetes and community members referred to their prevailing cultural meaning systems and schemas for infectious diseases to interpret and assign meaning to the emerging symptoms. Diabetes patients reported that they had initially used anti-malarial medicines because they believed their symptoms—like headache, fever, and tiredness—were suggestive of malaria. Schemas for body image informed the meaning given to diabetes symptoms similar to those of HIV, like severe weight loss. Confusion among members of the community about the diabetes symptoms instigated tension, causing patients to be mistrusted and stigmatized. The process of meaning-giving and the diagnosis of the diabetes symptoms was challenging for both patients and health care professionals. Diabetes patients reported being initially misdiagnosed and treated for other conditions by medical professionals. The inability to assign meaning to the symptoms and determine their etiologies informed the decision made by some patients to consult traditional healers, and to associate their symptoms with witchcraft causes.

**Conclusion:**

The meanings given to diabetes symptoms and the care-seeking practices described in the study are shaped by the prevailing cultural schemas for infectious diseases and their treatments. Efforts to educate people about the symptoms of diabetes and to encourage them to seek out appropriate care should build on the prevailing cultural meaning system and schemas for diseases, health and illness.

**Electronic supplementary material:**

The online version of this article (doi:10.1186/s12889-015-1504-0) contains supplementary material, which is available to authorized users.

## Background

The surge in the number of diabetes mellitus cases has become a major public health concern worldwide. There are currently around 382 million people with diabetes across the globe, and this figure is projected to rise to 592 million by 2035. An estimated 80% of these cases are in low- and middle-income countries (LMICs) [1]. For Sub-Saharan Africa (SSA), 14.7 million diabetes cases were reported in 2011. Thus, around 3.8% [2] of all people with diabetes are currently in this region, and the number of individuals with diabetes in SSA is expected to double by 2030 [3]. As in other countries currently undergoing the epidemiological transition, type 2 is the dominant form of diabetes in the SSA countries [4,5]. In Tanzania, more than one million people had diagnosed diabetes in 2006 [6] and the diabetes prevalence rate had reached 9.1% by 2012 [7]. However, it has been estimated that 80% of Tanzanians with diabetes are undiagnosed [8], possibly due to low levels of awareness and knowledge of the signs and symptoms of the disease [9-12]. Many people with diabetes likely die undiagnosed, and prematurely since they cannot survive long without treatment [9]. Diabetes is increasing in Tanzania at roughly equal rates among men and women and among urban and rural areas [7]. Currently, however, little is known about how diabetes is perceived by Tanzanians, including about the meanings people ascribe to the emerging symptoms, or the care-seeking practices of individuals with symptoms.

The increasing number of diabetes cases is placing additional burdens on the already strained health care systems in the majority of the SSA countries, including in Tanzania [13,14]. Meanwhile, communities with high diabetes rates are struggling to handle the accompanying social and the psychological challenges. The health care systems in these regions have been far more focused on combatting acute infectious conditions like malaria, tuberculosis, and HIV/AIDs [15,16] than on treating chronic non-communicable diseases (NCDs). These health services generally lack the types of medical supplies and laboratory equipment needed to diagnose and detect NCD conditions and their complications, and the levels of awareness of the signs and symptoms of these conditions remains low [9-12]. While dealing with infectious diseases remains a priority, addressing emerging NCDs is equally important.

Diabetes is a chronic and potentially life-threatening health condition [17]. When diabetes remains undiagnosed, untreated, or inadequately treated, it can develop into serious and potentially fatal conditions, including kidney failure, heart attack, stroke, ulcers requiring limb amputation, erectile dysfunction, and neuropathy [17]. Diabetes is also associated with increased susceptibility to other communicable [18,19] and non-communicable diseases [20,21]. The early detection of diabetes symptoms and the initiation of appropriate treatments can help to prevent or delay such complications. Whether people suffering from diabetes seek prompt and appropriate care depends in part, on how their communities interpret and ascribe meaning to the symptoms.

Across the globe, different communities have different traditions for interpreting and assigning meaning to symptoms when people are ill [22,23], which in turn affect the care-seeking practices of individuals [23]. How disease symptoms are interpreted, defined, and labeled is shaped by each community’s cultural meaning system [24-27]. According to D’Andrade [28], a community’s cultural meaning system consists of shared cultural schemas, including mental constructs for identifying and responding to new and familiar phenomena [28], such as diseases. These cultural schemas prescribe how people should act in or respond to specific situations [29,30], including being ill. These schemas are therefore the theoretical structures that facilitate the attribution of meaning to and the characterization of objects and actions. They “form the reality-defining system of the human and provide information about what states of the world can be and should be pursued” [28]). The wider community’s cultural schemas inform individuals’ beliefs, feelings, and perceptions regarding meaning in their lives. The schemas also shape people’s actions and experiences, including those related to health and illness, and thus to how they seek care when symptoms appear.

In this article, we use the cognitive anthropological perspectives of the cultural meaning system, or the “cultural schema,” in investigating the meanings given to the symptoms of diabetes in the communities studied, and how these meanings shape the care-seeking practices of individuals. Few existing studies have examined the meanings people attribute to the symptoms of emerging conditions—in this case, diabetes—and their initial efforts to seek care, especially in rural areas of countries undergoing the epidemiological transition, like Tanzania. The current study contributes to filling this research gap. Our findings can help to explain how the meanings assigned to disease symptoms in the community shape the care-seeking behaviors of individuals. Our results can also be used in shaping strategies for improving public awareness of diabetes symptoms which can encourage those with symptoms to seek prompt and appropriate care and help them to make informed decisions regarding the treatment and control of diabetes.

## Methods

### Study area and population

This study was conducted at the village of Viwanjasitini and at the diabetes clinic both in the district of Kilombero. This district has a population of 407,880, and is located in the southeastern part of Tanzania, 420 km from Dar es Salaam.

Viwanjasitini is a semi-urban village located 5 km from the diabetes clinic. The village has a total population of 12,823 (6,359 men and 6,464 women) residing in 2,175 households. The majority of the village residents have no more than a primary school education. Small-scale farming (primarily rice and maize) and animal husbandry (primarily pigs and chickens) are the villagers’ main economic activities. The results of our pilot study clearly showed that awareness and knowledge of the diabetes signs and symptoms were virtually non-existent in the general community.

The diabetes clinic, which is part of the designated district referral hospital, is the only clinic in the district that provides diabetes services primarily to people from rural and semi-urban areas. The inclusion of the clinic in the study provided us with an opportunity to recruit diabetes patients from rural areas and examine their care-seeking practices. The district faces a burden of infectious diseases such as Malaria [31,32] HIV and AIDS [33] and tuberculosis [34]. Whereas for non-communicable diseases such as diabetes the district also faces an increasing prevalence. An ongoing adult observational cohort study has reported that diabetes prevalence among women aged 60+ years in the district is 12% [35]. The current study was conducted concurrently with another study that assessed the care-seeking behaviors of adults with malaria; the results of this second study are presented separately [36].

### Study design and methods

To explore collective meanings assigned to diabetes symptoms in the community and by the people with diabetes we conducted nine focus group discussions (FGDs) with 58 adults (21 men and 37 women) between October and November 2012 (See Table 1). Of the 58 participants, 32 were people in the community and 26 were people with diabetes. To examine the care-seeking practices and how the meaning-giving system in the community shaped how individuals sought care we conducted 19 in-depth interviews with adult diabetes patients (10 women and 9 men) between February and March 2013.

**Table 1:** **Participants information for focus group discussions and in-depth interviews**

**Activity**	**Age range**	**Gender**		**Level of education**	**No. of people through the community**	**No. of people through the diabetes clinic**	**Total**
		**Male**	**Female**				
**FGDs**	25 - 80	21	37	0 – form IV	32	26	58
**IDIs**	25 - 80	9	10	0 – form IV	10	9	19
							77

### Participant recruitment

The FGDs with people in the community involved individuals who were purposively recruited from the village with the help of village leaders. These individuals were either neighbors or relatives of diabetes patients**.** Some FGDs also included people living with diabetes who were purposively recruited from the clinic. The recruitment of the participants for the FGDs ended when data saturation was reached.

All of the IDIs were conducted with diabetes patients who had been living with the condition for at least six months prior the date of the interview. The IDI participants from the semi-urban village were purposively selected from the community with the help of village leaders. We visited households in the community and asked whether anyone in the household had diabetes. To avoid recruiting people of the same social network, different entry points were used. The IDI participants at the diabetes clinic were purposively selected from the clinic attendees with the help of a clinic nurse. The researcher explained the purpose of her presence at the clinic to the patients and people with diabetes from rural areas were identified taking into consideration their age, gender, and years since diagnosis. After verbal consent was given interviews were conducted at a canteen located close to the designated district referral hospital. The recruitment of the IDIs participants ended after data saturation was reached. Some participants who came from rural areas were paid a small amount to help cover their transportation costs.

### Data collection and analysis

The data collection team consisted of the first author and two research assistants. The data collection guides (see Additional file 1) were piloted by the team, and the pilot results were used to refine the final guides employed in the study and to determine the FGD recruitment criteria. The actual data collection took place in two separate rounds. The team first conducted the FGDs. The results from the FGDs were used to improve the interview guides (see Additional file 2). The first author facilitated all of the discussions. One research assistant was engaged for note-taking in each of the FGDs. All of the FGDs and the IDIs were conducted in Swahili, a language spoken by the majority of people in the study communities and also by the first author. These discussions were digitally recorded and verbatim transcriptions were made within 48 hours of the time they were conducted.

The transcripts were reviewed and crosschecked for quality by the first author before they were imported to NVivo 9 (QSR International Pty Ltd, Australia). All of the transcripts were analyzed in their original language. The analysis process had two levels. The first level of analysis involved developing inductive and deductive codes and writing thick descriptions. Both the inductive and the deductive codes were developed by the first author. The codes were shared and discussed among the first three and the last author, and any discrepancies were reconciled among the four authors. The second level of analysis involved categorizing the codes into themes and family codes following principles of grounded theory. This paper presents results from the FGDs on the meanings assigned to diabetes symptoms in the community and the IDI findings on how these meanings shape the individual care-seeking practices of the diabetes patients. While the data provided by the patients’ FGDs and IDIs were fairly consistent, in the results section, these findings are not presented separately by method, but are instead used to describe different themes that emerge from the data.

### Ethical approval

The study was approved by institutional review boards of the Faculty of Spatial Sciences, University of Groningen in the Netherlands; the Ifakara Health Institute (IHI) in Tanzania; and the National Tanzanian Medical Research Co-coordinating Committee of the National Institute for Medical Research (NIMR). All participants were thoroughly informed of the study and then verbal consent was taken before the beginning of the IDIs and FGDs. Specific to the low-literacy setting verbal consent was the most suitable form of consent taking. To ensure anonymity of the study participants, all of their potential identifiers were removed from the data, and only their opinions are presented.

## Results

Five themes emerged from the data: (1) knowledge and awareness of diabetes signs and symptoms, (2) meanings given to diabetes symptoms, (3) the use of anti-malarial medicines (4) seeking diagnosis and treatment from health facilities, and (5) relatives’ advices and traditional healers’ consultations.

### Knowledge and awareness of diabetes signs and symptoms

Several symptoms of diabetes were mentioned in the study (see Table 2). In the group discussions with people from the community, addressing this issue sometimes required considerable probing. The diabetes patients reported having experienced one or several symptoms before they were diagnosed with the condition. However, none of these patients said they had initially understood that the symptoms they were experiencing were indicative of diabetes. Some of the diabetes symptoms mentioned by the study participants overlap with the general symptoms identified for diabetes by the International Diabetes Federation (IDF) [37] while others do not (see Table 2). Irrespective of their gender and place of residence, the study participants generally described diabetes as being a new disease in the community, and the majority of the people from the community appeared to lack knowledge and awareness of its signs and symptoms.

**Table 2:** **Diabetes symptoms as identified by the study participants and the International Diabetes Federation (IDF)**

**Symptoms identified by study participants**	**Symptoms identified by the IDF**
Frequent urination especially at night	Frequent urination
Extreme thirsty	Excessive thirst
Blurred vision	Blurred vision
Feeling hungry all the time	Increased hunger
Extreme tiredness	Tiredness
Severe loss of body weight	Weight loss
Loss of memory	Lack of interest and concentration
Numbness in the toes/feet	A tingling sensation or numbness in the hands or feet
Falling ill frequently	
Severe headache	
Being quick-tempered	
A dry mouth/throat	
Reduced body strength	
Excess sweating	
Pain all over the body	
Hallucinations	
Itching around genital areas	
High fever	
	Frequent infection
	Slow-healing wounds
	Vomiting and stomach pain

Most of the participants in the group discussions with people from the community reported having heard about “Kisukari”—meaning “a sugary disease”—but they could not describe its symptoms. Similar statements were made in the discussions with the diabetes patients. These patients reported that they had not recognized the condition before they were diagnosed. In the in-depth interviews, the patients said that when the symptoms started they did not associate them to diabetes because those symptoms were not familiar to them:

*“When it (diabetes) started I failed to define it because its symptoms were new*”^a^ Male patient, 75 yrs*.*

### Meanings given to diabetes symptoms

Some patients reported attributing some of the diabetes symptoms they experienced—like loss of strength, frequent urination, and excessive thirst—to witchcraft, because they could not assign meaning to the symptoms or explain their etiologies. Since diabetes was a relatively new disease in the community when the individuals started experiencing the condition, they drew upon their knowledge of infectious diseases from the existing cultural models and schemas in seeking to interpret and understand the new symptoms. When the prevailing cultural models for diseases in the community were not able to provide explanations for the symptoms they were experiencing, the patients often attributed their condition to external causes:

*“The feelings of being bewitched were completely there. You think …. for sure I have been bewitched, this must be witchcraft, it is not a disease from God. Those thoughts will always be there because first you are amazed that a person can fill a bucket in just a single night, and can drink so much water. I mean one bucket of water is not enough… you may even think you have been attacked by demons because that is not a normal condition”* Female patient, 52 yrs.

When the community members were interpreting and assigning meaning to the symptoms people with diabetes were experiencing, some attributed the symptoms to different diseases or to economic status. In the group discussions with people from the community, a number of participants said they see diabetes as a disease of rich people, as only the rich can afford to eat “good food” and sweet things. Interestingly, during the patient discussions, the participants noted that while diabetes was once seen as being “a disease of the rich,” it is starting to affect a majority of community members regardless of their wealth:

“*Previously they were calling it a disease of the rich, but nowadays it is a disease of all people”* Male Patient FGDs

It was spontaneously reported by participants in the study that diabetes patients are sometimes labeled as being HIV positive by members of the community because diabetes patients like HIV patients, often suffer a severe loss of body weight prior to treatment and are intermittently sick. The diabetes patients reported that people in the general community often doubted they had diabetes, and instead believed they were HIV positive. In elaborating on the meanings assigned to some of the symptoms diabetes patients’ experience, and how they are interpreted in the community, one of the participants in the patient group discussions reported having been confronted directly and asked:

“W*hy is your health so unstable… have you been tested* (for HIV)*?’ Even when you say I have been tested and I am just fine, you still hear:* ‘*You had better get another test at another place because sometimes they can miss it, if the second test shows you are ok, then you can be completely sure’*” Female patient FGDs.

The interpretations of and the meanings given to these symptoms were embedded in the community’s cultural meaning system regarding body image, and were specifically affected by the cultural value that a healthy person should look “fat.” The community’s system of assigning meanings to symptoms of disease appeared to inform the individual patients’ schemas and their own self-image:

*“…..this is not the body I had … I had heavy weight… so when I found my body weight decreasing day after day … I thought I had HIV/AIDS… actually many other people thought so as well*” Male patient, 42 yrs.

The above comment indicates that the patients’ interpretations of and the meanings they ascribe to their symptoms are shaped by their wider community’s cultural schema for infectious disease symptoms. Labeling people with diabetes as HIV positive creates tension in the community, and can cause them to be mistrusted and stigmatized. A number of the diabetes patients in the study said that being labeled HIV positive undermined the sense of trust between themselves and their family members. In the in-depth interviews, some of the patients reported that they had to undergo multiple HIV tests just to convince their relatives that they did not have AIDS:

“…*when I became thinner my mother said I must have been infected [*with AIDS*]. She told me to go to the hospital and find out; I told her I had taken all the tests, and that the tests showed that I am not infected. She said ‘I don’t believe you, let us go together.’ I had to go with her to the hospital. I was tested and I told them not to give me the results, but to instead give them to her. Another day there were experts walking through the streets testing for HIV/AIDS. I went and had another test and instructed them to give the results to my mother so she would be satisfied. When she got the second set of results she was finally satisfied.”* Female patient, 40 yrs

In describing the stigma diabetes patients face when they are labeled by the community as being HIV positive, the patients reported that they sometimes found that other people in the community were openly gossiping about and pointing fingers at them:

“*They were saying it openly… you find them sitting and saying ‘That one is infected she is not well’”* Female patient, 36 yrs

The high rates of infectious diseases in the community appear to have permeated people’s cultural meaning systems, leading them to interpret and label new disease symptoms accordingly. When the prevailing cultural frameworks and disease schemas were no longer useful in helping communities assign meanings to the symptoms observed, there was confusion among both the wider community and the specific individuals who experienced those symptoms. Even in cases in which the existing cultural frameworks were not able to explain the emerging conditions, the prevailing cultural schemas continued to be used, affecting the reactions of the individuals and of the community in general:

### Use of anti-malarial medicines

Because of the lack of familiarity with diabetes symptoms and the prevailing community schemas regarding body weakness and malaise, the emerging diabetes symptoms were often initially interpreted as being associated with malaria, a common febrile condition in the study area. A majority of the patients who were interviewed in-depth said that when they initially started feeling unwell they used malaria medicines, because they thought their symptoms—e.g., tiredness, fever, and headache—were caused by malaria. They explained that when these anti-malaria treatments did not alleviate their symptoms they sought additional care. This process reflects the cultural dominance of the infectious disease treatment framework in the wider community, which appears to have shaped the individuals’ care-seeking strategies:

“ *I was struggling ….Sometimes I bought malaria medicines from the shop and used them …When I found they did not help I decided to go to the health facilities… I went to a number of small dispensaries, trying every possible medicine but my condition remained the same… And this was before the sugar was diagnosed… Then I thought maybe I should go directly to the big hospital….. So I went there for testing.”* Male patient, 57 yrs

The above comment indicates that this patient was looking for a cure of the kind that might be expected when using treatments for infectious conditions such as malaria. The expectation that these treatments will provide a cure explains the patient’s care-seeking patterns. These practices mirror the existing cultural schema of the wider community regarding recovery from illness and the care-seeking practices associated with curable conditions. This situation might have informed the decisions of the individual patients, who likely were reluctant to assume that their symptoms were indicative of a non-infectious chronic condition. The idea of having a chronic and incurable condition did not seem to fit people’s cultural models of health and illness.

Some of the diabetes patients indicated that when they failed to properly interpret the symptoms and could not assign meaning to what they were experiencing, they became confused, and thought they probably had a disease that would clear up on its own. These patients reported that they then did nothing, used painkillers like “Panadol,” or drank plenty of water:

“*I did not have any dose… eeh.. my dose was just water and Panadol because I was confused…. I did not know what to do. I though probably it will end by itself but to my surprise it did not stop and I continued losing weight…. So I decided to go to the health facility.”* Female patient, 40 yrs

### Seeking diagnosis and treatment from health facilities

**Figure 1:**
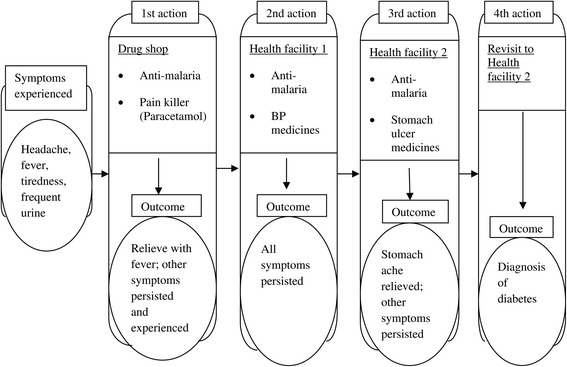
**A case study of the care seeking process for diabetes.**

The process of interpreting and assigning meaning to the diabetes symptoms, and then obtaining a diagnosis of the condition, was seen as challenging by the majority of participants in the study. A number of the diabetes patients said they faced problems in getting a diagnosis even after they sought care in modern health care facilities. During the patient group discussions, the majority of participants reported that when they went to the health facilities they were initially diagnosed and treated for other diseases, like urinary tract infections (UTIs) and sexual transmitted diseases (STDs), because of their frequent urination and itching around the genital area. Other patients said that when they visited the health facilities and explained to the clinician how they felt, a malaria test was ordered; and that when malaria was not detected they were sent home with painkillers only. Several of the patients reported that they were given a number of different tests before the provider finally prescribed a test for diabetes. This apparent tendency among health care workers to misdiagnose delayed the identification of the problem, and complicated the care-seeking responses of the patients and their ability to manage their condition. Figure 1 below depicts a case study of the process that one of the diabetes patients who was interviewed in-depth reported going through before she was finally correctly diagnosed.

The experience depicted in Figure 1 indicates that the prevailing schemas in the community for interpreting the symptoms of infectious diseases like malaria not only inform the care-seeking practices of individual community members; they also shape the initial responses of the health care workers to the emerging symptoms. This implies that among a majority of the health care workers responsible for diagnosing and treating patients, the cultural models of illness and the dominance of infectious diseases in the community shaped their initial interpretations of and the meanings they assigned to diabetes symptoms.

### Relatives’ advices and traditional healers’ consultations

Some of the patients who participated in the in-depth interviews reported that when their symptoms persisted due to misdiagnosis, they turned to friends and relatives for assistance in assigning meaning to the symptoms they were experiencing, and for advice on how to treat their condition. Some participants reported being advised to taste their own urine to see if it contained sugar:

*“I felt very thirsty, urinated frequently, and was drinking plenty of water. And when I urinated I would see ants following the trail. I did not understand what all this meant. I waited around 10 days and then I decided to go to a health facility to find out. I saw two doctors who worked there. I explained the problem to them and I had a blood test, but they did not diagnose it ….instead they gave me Panadol. I went home and saw one of my relatives. When I explained to him what happened he said … ‘This could be diabetes. If you want to find out take a few drops of your urine and taste for sugar.’ I followed his advice and tasted the sugar. I went back to the clinicians and told them that I have discovered I have diabetes. They asked, ‘How did you know?’ I said, ‘I tasted my urine.’ Then they referred me to the diabetes doctor….He ordered a test and found out I indeed have diabetes. Since then I have been having diabetes treatments.”* Male patient, 75 yr*s*

Some of these patients reported that when their relatives were not able to help them in interpreting or attributing meaning to their symptoms, or in labeling their condition or determining its etiology, their family members advised them to visit local healers for more help. The decision to visit local healers was described as being *a situation of rescuing life.* A participant in one of the group discussions with people in the community explained the appeal of traditional healers:

*“You know what causes all this [going to traditional healers] it is that people get sick and don’t get better… They think they have used medicines for malaria, typhoid and whatever …. but have seen little improvement. When they become thinner every day and their body continues to weaken then they want to try traditional healing. They think they must be bewitched or something like that. So one goes there [to traditional healers] in a situation of rescuing life.”* People in the community FGDs

Different reasons for consulting traditional healers were given in the focus group discussions with diabetes patients. A majority of participants in these discussions said people consult traditional healers in response to the symptoms they are experiencing, especially when they cannot interpret or ascribe meaning to their symptoms, or when health facility visits do not help them in interpreting the symptoms and diagnosing the problem. Others said that a visit to a traditional healer may be initiated when patients do not understand what is happening to their body, particularly when some of their symptoms are terrifying:

*“When I contracted diabetes I was very fat. I had a big belly, but it disappeared after only three days. I mean everyone who saw me was surprised, saying, ‘It has only three days!’ That is why I went to the traditional healer. I was afraid because I had changed a lot; it was a big change for just three days. I thought I was bewitched…”* Diabetes patient, FGDs

Although a number of participants reported consulting with traditional healers as a response to the diabetes symptoms they were experiencing, none of the participants claimed that these consultations provided a cure. The diabetes patients reported that when they visited the traditional healers they were diagnosed as being bewitched. Some of these patients said they used various concoctions, but continued to get worse. They then stopped seeing the healers and again sought help from health care facilities:

“*Honestly, after using all those medications I did not find relief in any of them… I was surprised… All of them* (the healers) *said I was bewitched…. I used lots of medications, but was surprised that the condition kept getting worse. That is when I thought, No let me just visit the health facility.*” Female patient, 58 yrs.

## Discussion

This study was conducted in order to provide insights into the meanings assigned to diabetes symptoms in the community, and how these meanings shape the care-seeking practices of individual patients. The results showed that among members of the community, awareness and knowledge of the diabetes signs and symptoms appear to be very limited, and people with diabetes knew little about the disease before they were diagnosed. These findings are in line with those of earlier studies for Tanzania [11] and other SSA countries [38-42]). The failure of individuals to correctly interpret, label, and assign meaning to their diabetes symptoms is indicative of the general lack of knowledge about diabetes in the community, and this has implications for the awareness of diabetes signs and symptoms and the care-seeking responses of patients. The low levels of awareness and knowledge of diabetes signs and its symptoms explain why patients were late in visiting health care facilities [43], and suggest that patients may find it difficult to recognize further risks associated with their condition.

As diabetes is relatively new to the community, the prevailing cultural meaning system for acute infectious diseases is used to interpret the symptoms, assign meaning, and label the condition. The study participants reported that diabetes symptoms are commonly misidentified as being those of malaria and HIV. The community’s cultural schema for body image—i.e., the assumption that a healthy body should look “fat”—appears to have informed this interpretation. The severe loss of body weight is a sign which is interpreted and imbued with meaning. The tendencies to ascribe certain meanings to diabetes symptoms and to interpret them as being those of HIV have also been reported in other African settings [44,45]. These perceptions can discourage undiagnosed individuals with diabetes from seeking care for fear they will be labeled HIV positive and stigmatized. The threat of social stigma can therefore have negative consequences for public health. Communities need to be informed about diabetes and its symptoms, and educated that a severe loss of body weight can be a sign of diabetes, as well as of HIV/AIDS.

The majority of the diabetes patients in the study reported having initially interpreted their symptoms as being indicative of malaria, a reported common illness in the district studied [46-48]. The misinterpretation of diabetes symptoms can lead to misdiagnosis and the inappropriate use of treatments, and can thus increase patients’ risks of developing long-term and potentially devastating complications [13,49]. The study found that both members of the community and health care professionals misinterpreted and misdiagnosed the symptoms of diabetes, as was observed during patient discussions. It led patients to be treated for other conditions. This may be the result of the prevailing cultural schemas for interpreting, diagnosing, and treating infectious illnesses that continue to dominate the health care systems in most of the SSA countries [5]. It also reflects the low levels of awareness of diabetes signs and symptoms among medical professionals and the poor quality of the diagnostic tools used by the majority of health facilities in Tanzania [11].

The majority of health care professionals in Tanzania are insufficiently equipped to diagnose and treat chronic diseases like diabetes, especially when working in rural and remote areas [11,50] where these conditions were once rare [16]. As chronic and lifelong conditions are becoming more prevalent among both rural and urban populations [7], it is important that health care professionals increase their knowledge and awareness of the signs and symptoms of these chronic conditions, and that the diagnostic capacity of health facilities be enhanced. In addition, the provision and delivery of health services in both urban and rural areas should be adapted to account for these emerging conditions.

It was the sweetness of their urine that helped patients to self-diagnose their condition, and people referred to diabetes as “Ki-sukari”, meaning a sweet disease. This is also known from other settings. For example, among Cameroonians, the cultural schemas and meanings attributed to diabetes have led into describing the condition as “a disease that is sweet” [51]. This description appears to have informed individuals’ responses to the condition and their subsequent care-seeking practices. In Ghana, diabetes, or “sugar disease,” is seen as a disease of wealthy people who can afford foods high in fat and sugar [52,53]. In Zimbabwe [54] and Uganda [44], the cultural explanations for and meanings ascribed to diabetes have often been related to supernatural forces, leading those suffering from the disease to seek care from traditional healers. These observations illustrate how cultural schemas for diseases and the meanings attributed to these conditions in the communities shape how individuals respond to their illness and seek care.

The inability of the community’s cultural meaning system to help individual patients in the study in interpreting their symptoms and giving meaning to what they were experiencing prompted these patients to attribute their symptoms to witchcraft. This finding is similar to observations made in Zimbabwe [54] and Uganda [44], in which diabetes patients reported they believed they were bewitched when they initially experienced symptoms. Attributing diabetes symptoms to witchcraft may lead patients to delay seeking appropriate care for their condition, and could result in complications.

The influence of family members and relatives on the care-seeking practices of patients with acute [55] and chronic [56] conditions has been widely reported. The patients interviewed in this study mentioned seeking care from traditional healers after being advised to do so by their relatives, especially when their family members could not help them in interpreting, labeling, and assigning meaning to their symptoms; and after they experienced no relief from their symptoms despite having visited a health care facility. Some study participants reported that they were motivated to seek out traditional healers because diabetes symptoms are associated with witchcraft. This finding is in line with the results of other studies for SSA which showed that healers are commonly consulted for conditions perceived as having witchcraft or spiritual associations [44,57]. These perceptions are part of the prevailing cultural schemas that inform the interpretation of illnesses, especially where the existing level of community knowledge cannot assign meanings and labels to the symptoms. In this study, patients reported consulting traditional healers when they could not make sense of the symptoms they were experiencing. As this was a conscious choice, this finding diverges from those of studies which showed that traditional healers were consulted mainly when conventional health services were unavailable or too far away [10,58]. Our results suggest there is a need to look at the role of traditional healers in interpreting and assigning meaning to diabetes symptoms, and in care-seeking behaviors. Programs that raise awareness in the community and involve traditional healers in encouraging people to visit conventional medical facilities have been shown to be successful in treating infectious conditions like malaria [59,60].

The care-seeking practices observed in this study reflect the fact that in Tanzania acute infectious diseases such as malaria have been dominant, while chronic diseases have been relatively rare. Our study results indicate that prior to diagnosis the diabetes patients were expecting to receive medication that would ameliorate their symptoms and cure them. This tendency to search for a cure for their symptoms explains the care-seeking patterns observed in the study. Diabetes is a chronic and lifelong condition that cannot be cured, but which can be treated to minimize its effects and prevent potentially devastating complications [17,61]. Community members need to be informed about the difference between chronic and acute illnesses.

This study provides very important findings on the initial responses to the appearance of diabetes symptoms among people living in rural communities. However, as the study was restricted to a particular group of people who were purposively selected, the results can only be generalized to settings with a similar context. Although these findings are specific to the site of the study, it is likely that similar processes exist in other parts of Tanzania. However, the results of this study could be used as a basis for a larger survey which could attempt to confirm them in a broader setting.

## Conclusion

This study showed that there is a lack of awareness about diabetes signs and symptoms in the communities investigated. As a consequence, the prevailing cultural schemas for infectious diseases in the community influenced the interpretation of diabetes symptoms by both patients and health professionals and shaped the care-seeking practices of individual patients. The misdiagnosis of diabetes led patients to use anti-malarial medications, to consult traditional healers, and to attribute their diabetes symptoms to witchcraft. In educating communities about emerging chronic diseases, building on the prevailing cultural meaning system and schemas for diseases, health, and illness is recommended.

## Endnote

^a^As all of the quotes have been translated from Swahili to English, they may not follow a strict grammatical route.

## Additional files

Additional file 1:
**Focus group discussion topic guides on diabetes.**
Additional file 2:
**In-depth interview topic guides on diabetes.**

## Abbreviations

AIDs: Acquired immunodeficiency syndrome
FGD: Focus group discussion
HIV: Human immunodeficiency virus
IDI: In-depth interviews
LMICs: Low and middle income countries
NCDs: Non communicable diseases
SSA: Sub-Saharan Africa
STD: Sexual transmitted diseases
UTI: Urinary truck infections

## References

[1] IDF: IDF Diabetes Atlas, 6th edn., I.D. Federation, Edition 2013, International Diabetes association: Brussels, Belgium 2013. http://www.idf.org/sites/default/files/EN_6E_Atlas_Full_0.pdf. Accessed on 20^th^ February, 2014.

[2] IDF: IDF diabetes Atlas, 5th edn., I.D. Federation, Edition 2011, International Diabetes association: Brussels, Belgium 2011 http://www.idf.org/diabetesatlas/5e. Accessed 15^th^ October 2012.

[3] Shaw J, Sicree R, Zimmet P (2010). Global estimates of the prevalence of diabetes for 2010 and 2030. Diabetes Res Clin Pract.

[4] Mbanya JC, Kengne AP, Assah F (2006). Diabetes care in Africa. Lancet.

[5] Azevedo M, Alla S (2008). Diabetes in sub-saharan Africa: Kenya, Mali, Mozambique, Nigeria, South Africa and Zambia. Int J Diabetes Dev Ctries.

[6] Bakker K, Abbas Z, Pendsey S (2006). Step by Step, improving diabetic foot care in the developing world. Pract Diabetes Int.

[7] WHO: WHO TANZANIA STEPS Survey-2012 Fact sheet. http://www.who.int/chp/steps/UR_Tanzania_FactSheet_2012.pdf Accessed on 12th August, 2013.

[8] Aspray TJ, Mugusi F, Rashid S, Whiting D, Edwards R, Alberti KG (2000). Rural and urban differences in diabetes prevalence in Tanzania: the role of obesity, physical inactivity and urban living. Trans R Soc Trop Med Hyg.

[9] Simpson K (2003). Diabetes in Tanzania: insulin supply and availability. J R Coll Physicians Edinb.

[10] Simpson K (2007). Use of alternative medicine in patients with diabetes in Tanzania. Pract Diabetes Int.

[11] Ramaiya K (2005). Personal view: Tanzania and diabetes—a model for developing countries?. BMJ Br Med J.

[12] Kolling M, Winkley K, Von Deden M (2010). Research“ For someone who’s rich, it’s not a problem”. Insights from Tanzania on diabetes health-seeking and medical pluralism among Dar es Salaam’s urban poor. Global Health.

[13] Mbanya JCN, Motala AA, Sobngwi E, Assah FK, Enoru ST (2010). Diabetes in sub-Saharan Africa. Lancet.

[14] Beran D, Yudkin JS (2006). Diabetes care in sub-Saharan Africa. Lancet.

[15] Assah FK, Mbanya J-C (2009). Diabetes in sub-Saharan Africa-overview of a looming health challenge. Eur Endocrinol.

[16] Hall V, Thomsen RW, Henriksen O, Lohse N (2011). Diabetes in Sub Saharan Africa 1999–2011: epidemiology and public health implications. A systematic review. BMC Public Health.

[17] The hidden pandemic and its impact in Sub Saharan Africa. Diabetes Leadership Forum Africa. http://www.changingdiabetesbarometer.com/docs/Diabetes%20in%20sub-saharan%20Africa.pdf accessed on 23rd Dec 2013.

[18] Danquah I, Bedu-Addo G, Mockenhaupt FP (2010). Type 2 diabetes mellitus and increased risk for malaria infection. Emerg Infect Dis.

[19] Faurholt-Jepsen D, Range N, PrayGod G, Jeremiah K, Faurholt-Jepsen M, Aabye MG (2011). Diabetes is a risk factor for pulmonary tuberculosis: a case–control study from Mwanza, Tanzania. PLoS One.

[20] Saydah SH, Eberhardt MS, Loria CM, Brancati FL (2002). Age and the burden of death attributable to diabetes in the United States. Am J Epidemiol.

[21] Brown WV (2008). Microvascular complications of diabetes mellitus: renal protection accompanies cardiovascular protection. Am J Cardiol.

[22] Helman CG (2007). Culture, Health and Illness. Arnold, Hodder Headline Group.

[23] Hedemalm A, Schaufelberger M, Ekman I (2008). Symptom recognition and health care seeking among immigrants and native Swedish patients with heart failure. BMC Nursing.

[24] Boonmongkon P, Streefland P, Tan M (2001). Applied health research manual: anthropology of health and health care.

[25] Paniagua FA, Paniagua F (2000). Handbook of multicultural mental health: Assessment and treatment of diverse populations.

[26] MacLachlan M (2006). Culture and health: A critical perspective towards global health.

[27] Edman JL, Kameoka VA (1997). Cultural differences in illness schemas an analysis of filipino and american illness attributions. J Cross Cult Psychol.

[28] D’Andrade RG, Strauss C (1992). Human motives and cultural models.

[29] Banerjee S (2006). Higher Education and the Reproductive Life Course: A Cross-cultural Study of Women in Karnataka (India) and the Netherlands.

[30] Hutter I, Ramesh B. The role of cultural schemas and cultural meaning systems regarding demographic and reproductive health behaviour in South India. In Paper presented to the Population Association of America Annual Meeting May 1–3, 2003 Minneapolis.

[31] Drakeley C, Schellenberg D, Kihonda J, Sousa C, Arez A, Lopes D, et al. An estimation of the entomological inoculation rate for Ifakara: a semi-urban area in a region of intense malaria transmission in Tanzania. Tropic Med Int Health. 2003;8:767–74. 10.1046/j.1365-3156.2003.01100.x 12950662

[32] Hetzel M, Dillip A, Lengeler C, Obrist B, Msechu J, Makemba A, et al. Malaria treatment in the retail sector: knowledge and practices of drug sellers in rural Tanzania. BMC Public Health. 2008;8:157. 10.1186/1471-2458-8-157 PMC2405791 18471299

[33] Nombo CI (2007). When AIDS meets poverty: implications for social capital in a village in Tanzania.

[34] Lwilla F, Schellenberg D, Masanja H, Acosta C, Galindo C, Aponte J (2003). Evaluation of efficacy of community based vs. institutional based direct observed short course treatment for the control of tuberculosis in Kilombero district, Tanzania. Tropic Med Int Health.

[35] Eveline Geubbels RA, Sally M, Rollanda F, Khamis A, Tumaini K, Mary M, et al. High community prevalence of non-communicable disease and risk factors in Ifakara DSS, rural Tanzania. In: 12th Indepth Scientific Conference 2013: 28th - 31st October 2013; Johannesburg, South Africa. 2013

[36] Metta E, Haisma H, Kessy F, Hutter I, Bailey A (2014). “We have become doctors for ourselves”: motives for malaria self-care among adults in southeastern Tanzania. Malar J..

[37] Signs and Symptoms of Diabetes. http://www.idf.org/signs-and-symptoms-diabetes. Accessed on 31 January 2014.

[38] Gunay T, Ulusel B, Velipasaoglu S, Unal B, Ucku R, Ozgener N (2006). Factors affecting adult knowledge of diabetes in Narlidere Health District, Turkey. Acta Diabetol..

[39] Murugesan N, Snehalatha C, Shobhana R, Roglic G, Ramachandran A (2007). Awareness about diabetes and its complications in the general and diabetic population in a city in southern India. Diabetes Res Clin Pract..

[40] Al Shafaee MA, Al-Shukaili S, Rizvi SG, Al Farsi Y, Khan MA, Ganguly SS (2008). Knowledge and perceptions of diabetes in a semi-urban Omani population. BMC Public Health..

[41] Maina WK, Ndegwa ZM, Njenga EW, Muchemi EW (2011). Knowledge, attitude and practices related to diabetes among community members in four provinces of Kenya: A cross-sectional study. J Pan Afr Med.

[42] Hamoudi NM, Al Ayoubi ID, Al Sharbatti S, Shirwaikar A (2012). Awareness of diabetes mellitus among UAE non-diabetic population in Ajman and Ras Alkhaimah. J Appl Pharm Sci..

[43] Echouffo-Tcheugui JB, Mayige M, Ogbera AO, Sobgnwi E, Kengne AP (2012). Screening for hyperglycemia in the developing world: rationale, challenges and opportunities. Diabetes Res Clin Pract.

[44] Rutebemberwa E, Katureebe SK, Gitta SN, Mwaka AD, Atuyambe L, Network K (2013). Perceptions of diabetes in rural areas of Eastern Uganda. Curationis..

[45] Gill G, Mbanya J-C, Ramaiya K, Tesfaye S (2009). A sub-Saharan African perspective of diabetes. Diabetologia..

[46] Hetzel MW, Obrist B, Lengeler C, Msechu JJ, Nathan R, Dillip A (2008). Obstacles to prompt and effective malaria treatment lead to low community-coverage in two rural districts of Tanzania. BMC Public Health..

[47] Smithson P (2009). Down but not out. The impact of malaria control in Tanzania. Ifakara Health Institute Spotlight.

[48] Schellenberg D, Menendez C, Kahigwa E, Font F, Galindo C, Acosta C (1999). African children with malaria in an area of intense Plasmodium falciparum transmission: features on admission to the hospital and risk factors for death. AmJTrop Med Hyg.

[49] Dagogo-Jack S (2006). Primary prevention of type-2 diabetes in developing countries. J Natl Med Assoc.

[50] MoHSW. “Tanzania Service Availability and Readiness Assessment (SARA) 2012” Ifakara Health Institute, Dar es Salaam. Dar es Salaam, Tanzania, 2013.

[51] Awah PK, Unwin NC, Phillimore PR (2009). Diabetes Mellitus: Indigenous naming, indigenous diagnosis and self-management in an African setting: the example from Cameroon. BMC Endocr Disord.

[52] Aikins AD-G (2003). Living with diabetes in rural and urban Ghana: a critical social psychological examination of illness action and scope for intervention. J Health Psychol.

[53] Aikins AD-G (2004). Strengthening quality and continuity of diabetes care in rural Ghana: a critical social psychological approach. J Health Psychol.

[54] Hjelm K, Mufunda E (2010). Zimbabwean diabetics’ beliefs about health and illness: an interview study. BMC Int Health Hum Rights.

[55] Nyamongo I (2002). Health care switching behaviour of malaria patients in a Kenyan rural community. Soc Sci Med.

[56] Mshana G, Hampshire K, Panter-Brick C, Walker R (2008). Urban–rural contrasts in explanatory models and treatment-seeking behaviours for stroke in Tanzania. J Biosoc Sci.

[57] Comoro C, Nsimba S, Warsame M, Tomson G (2003). Local understanding, perceptions and reported practices of mothers/guardians and health workers on childhood malaria in a Tanzanian district—implications for malaria control. Acta Trop.

[58] Pal SK (2002). Complementary and alternative medicine: an overview. Curr Sci Banglore.

[59] De Savigny D, Mayombana C, Mwageni E, Masanja H, Minhaj A, Mkilindi Y (2004). Care-seeking patterns for fatal malaria in Tanzania. Malar J.

[60] Dillip A, Hetzel MW, Gosoniu D, Kessy F, Lengeler C, Mayumana I (2009). Socio-cultural factors explaining timely and appropriate use of health facilities for degedege in south-eastern Tanzania. Malar J.

[61] Funnell MM (2010). Peer-based behavioural strategies to improve chronic disease self-management and clinical outcomes: evidence, logistics, evaluation considerations and needs for future research. Fam Pract.

