# Supplementary material for: “In a situation of rescuing life”: meanings given to diabetes symptoms and care-seeking practices among adults in Southeastern Tanzania: a qualitative inquiry
Source: BMC Public Health. 2015 Mar 7;15:224. doi: 10.1186/s12889-015-1504-0 (PMC4358854; doi:10.1186/s12889-015-1504-0)
Supplement: Additional file 1: — Focus group discussion topic guides on diabetes. [file 12889_2015_1504_MOESM1_ESM.doc]

**Additional file1: FOCUS GROUP DISCUSSION TOPIC GUIDES ON DIABETES**

**Introduce yourself to the group and explain again the following:**

- ***Explain the general purpose of the study***: To learn about the cultural aspects influencing health seeking behavior for diabetes
- ***Aim of the discussion:*** To learn about diabetes and its treatment practices
- ***Expected discussion time***: Aproximately1:30 hours
- ***Why the participant’s cooperation is important***: Your participation and cooperation is very important for the success of this study as we would like to learn from you; your views, opinions and experiences with diabetes and health seeking behavior in general.
- **What if participants don’t want to be part of the study**? Being part of this study is important but it is up to you to decide. If you do not want to take part in this study, it will not affect any care or treatment you receive. It will not cost you or your family anything.
- ***What will happen with the collected information and how the study participants will benefit***: The information collected will be used only for the intended purpose of meeting the academic requirements of the PhD candidate. This information will not help you directly, but it could benefit many other people in future because it will help the program managers and policy makers to improve their diabetic control programs
- **Confidentiality**: The information that we will discuss here today will remain confidential and no one apart from the research team will have access to these data. Your names will be removed from the data and no one will be able to link your name with what is said. This data will be published and shared with the scientific community but your name will not appear in any of these publications
- ***Use of tape recorder:*** To be able to keep a more accurate record of our discussion I am proposing to use a tape recorder if you don’t mind. Do you mind if I use a tape recorder? *(observe whether people agrees)*
- Do you have any question?
- ***Consent***: Do you agree to take part in this discussion? (observe whether people agrees)
- The moderator turn on the digital recorder and start the discussion

***Note: Group discussants background characteristics fill appendix Ai – FGDs participants’ recruitment form. (Remember some of the aspects in this form are to be filled at the end of discussion – example participants age and education level)***

1. Let us start our discussion by talking about illnesses in this /your village. What are the illnesses found in this /your village? Probe:

a. What are the illnesses affecting adult people more? probe for all illness affecting adults in the community.

1. ***If not mentioned****:* Ask if have ever heard of diabetes? Probe:
2. What do they know about it?
3. Where have they heard about?
4. What do they hear about it?
   1. What is the cause of diabetes? (Probe: all possible causes? can one get diabetes from another person?)
   2. How is diabetes treated?
   3. How can people prevent themselves from getting diabetes? (Probe: for all possible measures that people can take)
5. How is diabetes known in this /your village? Probe:
   1. What is the local name for diabetes?
   2. How is diabetes associated with other diseases in the village? (Probe: what are those diseases? And what are the reasons for such association?)
6. How can you describe the issue of diabetes in this /your village? Is it an issue of concern? Why? Probe:
7. Are there many people with *local name if available* (diabetes) in the village
8. Who gets *local name if available* (diabetes) in the village? Probe also in terms of gender and age?
9. Why is it that those people (mentioned) are getting *local name if available* (diabetes) and not others?
10. Is it possible for anyone to get *local name if available* (diabetes)? Why?
11. How do people know when they have diabetes? Probe: symptoms that identify presence of diabetes and which ones are most common?)
12. What do people do when they think they have *local name if available* (diabetes) in this /your village? Probe:
13. For different actions that people take and why they do so? e.g self treatment and why? going to health facility – of which type and why? drug shop and why? use of traditional/local medicines and why? How is it if diabetic people will not take any action concerning their health? How is this happening in the village?
14. What comes up to your mind when you think of going for diabetes treatments in the health facilities? Probe for:
    1. Medication costs – consultation and medicine costs
    2. Availability of medicines
    3. What about health care workers
    4. Waiting time before consultations
    5. What can you say of the whole process of seeking diabetes care at the health facility?
15. Where else can people get treatments for diabetes in this/your village? What type of treatment is obtained there? Probe: use of traditional healers; when traditional healers are consulted; when health facility is consulted and why?
16. How is the life of diabetes people in this/your village? Probe:
    1. Their participation in social gatherings?
    2. Their workings in the farms/or other activities?
    3. What about foods? Why?
    4. What about life style change prescriptions? Why?
    5. In continuing with their medications? Why?
17. What are your opinions concerning the use of diabetes treatments prescriptions? (Probe: Is there a need for people with diabetes to continue with their medical prescriptions? Why? Probe for both continuing medicine use and practicing life style prescriptions)
18. Who are involved in helping *local name if available* (diabetes) people in seeking treatment in this/your village? (Probe: relatives of the sick person, family members, friends or any villager/neighbors etc) Why are those people involved?
19. What support do people with *local name if available* (diabetes) need from their family and friends in taking care of their illnesses properly?
20. What are the challenges faced by families as a result of having *local name if available* (diabetes) people? Why? Probe for social, economic and health challenges
21. What do you think needs to be done for people with *local name if available* (diabetes) to seek diabetes treatments promptly and to continue with their medical prescriptions? (probe for both continue with treatments and practice life style changes. Who is to do it? What can people themselves do?)
22. What can be done for family members or caregivers to be able to help people with *local name if available* (diabetes) to continue well with their medical prescriptions? Probe: treatment use continuity and life style prescriptions?
23. Are there other issues about *local name if available* (diabetes) that we have forgotten and you would like to share with us? What are they?

**THANK YOU FOR YOUR TIME AND COOPERATION**
