# Supplementary material for: “In a situation of rescuing life”: meanings given to diabetes symptoms and care-seeking practices among adults in Southeastern Tanzania: a qualitative inquiry
Source: BMC Public Health. 2015 Mar 7;15:224. doi: 10.1186/s12889-015-1504-0 (PMC4358854; doi:10.1186/s12889-015-1504-0)
Supplement: Additional file 2: — In-depth interview topic guides on diabetes. [file 12889_2015_1504_MOESM2_ESM.doc]

**Additional file2: TOPIC GUIDE FOR DIABETES INDEPTH INTERVIEW**

**Introduce yourself to the participant and explain again the following:**

- ***Explain the general purpose of the study***: To learn about the cultural aspects influencing health seeking behavior for diabetes
- ***Aim of the interview:*** To learn about diabetes and its treatment practices
- ***Expected interview time***: Approximately 1 hour
- ***Why the participant’s cooperation is important***: Your participation and cooperation is very important for the success of this study as I would like to learn from you; your views, opinions and experiences with diabetes and health seeking behavior in general.
- **What if a participant doesn’t want to be part of the study**? Being part of this study is important but it is up to you to decide. If you do not want to take part in this study, it will not affect any care or treatment you receive. It will not cost you or your family anything.
- ***What will happen with the collected information and how the study participants will benefit***: The information collected will be used only for the intended purpose of meeting the academic requirements of the PhD candidate. This information will not help you directly, but it could benefit many other people in future because it will help the program managers and policy makers to improve their diabetic control programs
- **Confidentiality**: The information that we will discuss here today will remain confidential and no one apart from the research team will have access to these data. Your names will be removed from the data and no one will be able to link your name with what is said. This data will be published and shared with the scientific community but your name will not appear in any of these publications
- ***Use of tape recorder:*** To be able to keep a more accurate record of our discussion I am proposing to use a tape recorder if you don’t mind. Do you mind if I use a tape recorder? *(observe whether people agrees)*
- Do you have any question?
- ***Consent***: Do you agree to take part in this discussion? (observe whether people agrees)
- The moderator turn on the digital recorder and start the discussion

**Section A: Background characteristics:**

1. I would like to know more about you and your family:
2. What is your age? ______________________________
3. What is your tribe? ___________________________________
4. What is your religion?________________________
5. Have you been to school? If yes, up to which level?__________________
6. What is your source of income? ________________
7. Are you married?___________________________
8. What is you spouses education level?_____________________
9. What is your spouse’s source of income? ____________________
10. How many children do you have? ________________________________
11. How long since you have been diagnosed with diabetes?______________
12. Perhaps, you could now tell me a little bit about your family members, who do you live with? And how do you relate with them? (*For recorder:* Names are not important but give number and fill in the table below):

| S/N | Relationship | Age | Gender | Religion | Education level | Source of income |
| --- | --- | --- | --- | --- | --- | --- |
| *eg* | *Aunt* | *20yrs* | *Female* | *Christian* | *Std 7* | *Petty trader* |
| 1 |  |  |  |  |  |  |
| 2 |  |  |  |  |  |  |
| 3 |  |  |  |  |  |  |
| 4 |  |  |  |  |  |  |
| 5 |  |  |  |  |  |  |
| 6 |  |  |  |  |  |  |

**Section B: Health seeking behavior practices**

1. As you already told me that you are having diabetes could you please tell me a little more about this illness?

- 1. How it started?
  2. What were the initial symptoms you had? Probe: Are you still experiencing the same symptom?
  3. What was the first thing you did after noticing the symptoms? Why? (Probe: for each action taken and reasons for doing so)
  4. Who was with you or helped you decide on the actions you took? Why did you involve this person?

2. What do you think is the cause of the diabetes you are suffering from? Probe:

- 1. All possible causes and why?
  2. Do you think one can get diabetes from another person?
  3. How can one protect or prevent him/herself from getting diabetes?
  4. How is diabetes cured? Probe: is it a curable disease? Why?

1. Before you were diagnosed with diabetes, did you know that you might have been suffering from it? (Probe: If yes, how did you know? If no, what prompted you to seek care?)
2. How did you feel when you were first diagnosed with diabetes? Probe:
   1. Was this the first time to hear about diabetes or you already heard about it?
   2. Where did you hear about it? And what information did you have?
   3. What did you do when you were first diagnosed with diabetes? Probe: did you tell your family/friends and other people about it? Why?
   4. In your opinion, what do other people in the family think about you because of having diabetes? Why?
3. How long did it take between the time you noted the diabetes signs or symptoms and the time you decided to seek care at the health facility? Why?
4. What treatments did you use before you went to the health facility? (Probe according to the type of treatment used):
   1. Self medication – what type of medicines were used? How did you get them? Why self treated first? Who gave the advice?
   2. Bought medicine from drug shop – what type of medicine, who advised that medicine? Why buying drugs from the shop before hospital consultation?
   3. Use of traditional herbs – which ones were used? How did you get them? Who advised their use?
5. How was it then you went to the health facility after using medicines you just mentioned? Probe:
   1. What prompted his/her use of the health facility services
   2. Who was involved in making decisions for seeking diabetes treatments
6. What support do you need from your family or friends to be able to seek treatment for your diabetes? (Probe: the type of support and who is giving it)
7. What preparations do you have to make before going to seek care from the health facility?

Probe: for medical costs (consultation and treatment costs); distance/transportation costs; and how they affect his/her decision to seek care?

1. Tell me a little more on the processes of seeking diabetes care at the health facility?
   1. How long do you have to wait before seeing the specialist?
   2. What about medical costs – consultation and treatment costs?
   3. How long did you go with the same symptoms before being diagnosed with diabetes?
   4. What instructions did the health care worker gave you on using medications? Do you understand well? How can you describe your relationship with the health care worker?
   5. What is your opinion concerning the way consultations are made? How would you like it to be
2. Tell me a little more about diabetes self-treatments at home? What challenges are you facing? (Probe: How does cost of care and treatments affect your continuity with the use of the recommended treatment?)
3. Can you please explain again a little more on how you feel about the life style changes prescriptions? Probe: How are you managing with the life style changes prescriptions? What are the challenges faced and how do you cope with them?
4. How does the cost of care and treatment for diabetes affect the welfare of your family? (Probe: How do you cope with this problem?)
5. How do you feel about the diabetes treatments that you are using? (Probe: for personal experiences and feelings on the medicines used and lifestyle change practices; How is it helping you? Do you think there is need to continue using as was advised? )
6. Now let us talk a little more about the use of traditional healer’s services for diabetes treatments, what is your experience on this? (Probe: have you ever sought traditional services for your diabetes? What made you to do so? What kind of services did you get?)
7. Where is the other place that you went to seek care after initiating treatment from the health facility/diabetes clinic? What type of service did you get? And what are the reasons for doing that? – Probe for any type of care eg prayers etc
8. What consequences one can get as results of having diabetes? Probe for: social, economic and health consequences
9. What do you think need to be done to help people with diabetes attend their diabetes clinic as required? Probe:
   1. What can health care workers do to help people with diabetes to comply better with their medical regimen? – probe for both continuity with treatments and life style changes
10. What support do you get from your family and friends in taking care of your diabetes? (Probe: Who is providing most of your care/support and what are the tasks that this caregiver carries out for you?
11. What do you think needs to be done for patient caregivers/family members to provide care to people with diabetes?
12. Is there anything concerning diabetes that we have left /missed and you would like to share with me? What is it?

**THANK YOU FOR YOUR TIME AND COOPERATION**
